# Antioxidant System Response of *Yarrowia lipolytica* Cells Under Oxidative Stress

**DOI:** 10.3390/ijms26199629

**Published:** 2025-10-02

**Authors:** Gerardo Ismael Arredondo-Mendoza, Maripaz Castillo-Roque, Hipólito Otoniel Miranda-Roblero, María Fernanda Desentis-Desentis, Sandra Lucía Teniente, Zacarías Jiménez-Salas, Eduardo Campos-Góngora

**Affiliations:** Universidad Autónoma de Nuevo León, Centro de Investigación en Nutrición y Salud Pública, Facultad de Salud Pública y Nutrición, Av. Dr. Eduardo Aguirre Pequeño y Yuriria, Col. Mitras Centro, Monterrey CP. 64460, Nuevo León, Mexico; gerardo.arredondom@uanl.mx (G.I.A.-M.); maripaz.castillorq@uanl.edu.mx (M.C.-R.); otto.miranda@tec.mx (H.O.M.-R.); fernanda_dd@hotmail.com (M.F.D.-D.); steniente@uadec.edu.mx (S.L.T.); zacarias.jimenezs@uanl.mx (Z.J.-S.)

**Keywords:** oxidative stress, antioxidant response, gene expression, *Yarrowia lipolytica*

## Abstract

Eukaryotic cells respond to oxidative stress (OS), a physiological condition characterized by the accumulation of reactive oxygen species (ROS), through various protective mechanisms. The antioxidant defense system (ADS) is activated either by post-translational modifications of pre-existing proteins or through the induction of gene expression. These mechanisms protect cellular biomolecules against ROS damage. Although extensive research has been conducted in different species, there is limited information regarding the specific response of *Yarrowia lipolytica* to OS. This study aims to elucidate the molecular mechanisms by which *Y. lipolytica* responds to OS by analyzing the expression of genes encoding enzymes involved in antioxidant response, such as superoxide dismutase (Sod), catalase (Cat), and glutathione peroxidase (Gpx). The *Y. lipolytica* genome contains three *CAT* genes, one *SOD* gene, one copper chaperone for Sod (*CCS*) gene, and one *GPX* gene. The expression profiles of these genes were assessed in *Y. lipolytica* cells exposed to H_2_O_2_ [5 mM] over time. All genes reached their maximal expression within the first 15 min of exposure. Comparative analysis between young and aged *Y. lipolytica* cells subjected to OS revealed that young cells exhibited higher expression levels for all genes, with *CAT3* and *SOD* showing the highest expression values. These findings suggest that the enzymes encoded by these genes play a crucial role in the antioxidant response of this species. To our knowledge, this is the first study demonstrating that the ADS in *Y. lipolytica* is regulated at the transcriptional level.

## 1. Introduction

Oxidative stress (OS) is defined as an imbalance between the production and degradation of reactive oxygen species (ROS), a term used to collectively designate oxygen radicals that behave as oxidants [1,2,3,4,5]. Molecular forms of ROS include superoxide (O_2_^−^), perhydroxyl (HOO^−^), hydroxyl (HO^−^), peroxyl (ROO^−^), and alkoxyl (RO), as well as non-radical species such as hydrogen peroxide (H_2_O_2_), singlet oxygen (^1^O_2_), hypochlorous acid (HOCl), and organic hydroperoxide (ROOH) [6,7]. ROS play key roles in several signaling pathways critical for cellular function. However, when the balance between ROS levels and antioxidant enzymes is disrupted, ROS can interact with macromolecules causing cellular damage and contributing to the development of OS [6,8,9]. ROS accumulation also increases during cellular aging [6,10,11,12]. In aging cells, the loss of division capacity, telomere shortening, DNA damage, mitochondrial dysfunction, and subsequent apoptosis can occur [13]. Since ROS are involved in an essential metabolic process in aerobic organisms, their production is inevitable and, although ROS are produced under normal physiological conditions, they can damage cellular structures, including lipids, proteins, and nucleic acids [14,15]. Damage to biomolecules, along with the induction of pro-inflammatory cytokine expression and activation of signaling pathways by OS are associated with aging and several pathological conditions, such as cancer, atherosclerosis, neurodegenerative diseases, obesity, and diabetes [11,14,16,17]. In eukaryotic cells, the electron transport chain is the primary endogenous source of ROS; other endogenous sources of free radicals include the protein folding process, disulfide bonds’ formation in the endoplasmic reticulum, cytochrome P450-mediated metabolism of xenobiotics, and the activity of neutrophils, macrophages, and eosinophils [1,11,14,18,19,20]. Biological systems counteract ROS damage through an array of enzymatic and non-enzymatic molecules that comprise the antioxidant defense system (ADS). The ADS consists of two kinds of molecules: those synthesized by a cell itself (endogenous) and those obtained from external sources (exogenous) [1,5,21,22].

In both prokaryotic and eukaryotic cells, the endogenous ADS operates through enzymatic reactions involving three primary enzymes: superoxide dismutase (Sod), which catalyzes the dismutation of superoxide radicals into oxygen and H_2_O_2_; catalase (Cat), which catalyzes the breakdown of H_2_O_2_ into oxygen and water; and glutathione peroxidase (Gpx), which reduces H_2_O_2_ using glutathione [1,18,21,23]. These antioxidant enzymes are encoded by *SOD*, *CAT*, and *GPX* genes, respectively. In cells of the yeast *S. cerevisiae*, the expression of these “antioxidant genes” is regulated at the transcriptional level by transcription factors such as Yap1 and Skn7 in response to H_2_O_2_ [24]. Yap1 is a major regulator of the ADS in *S. cerevisiae* which is distributed mainly in the cell cytosol, whereas Skn7 is a constitutive nuclear protein that functions in combination with Yap1 in response to peroxides. In addition, some genes controlled by Yap1 can be regulated by both Yap1 and Skn7 transcription factors [24].

Recent studies in yeast models have led to the identification of genetic determinants involved in aging process. These cellular models offer advantages due to their short lifespan and ease of genetic manipulation [25,26]. Yeast shares highly conserved metabolic and signaling pathways with human cells. In addition, many of the genes involved in lifespan regulation, damage repair, and OS responses in yeasts have human homologs [25,26,27,28,29].

In recent years, *Yarrowia lipolytica*—considered a non-conventional yeast—has been widely used as a biological model to study different biological and biotechnological processes including cell morphogenesis, differentiation, secretion of heterologous proteins, and aging [26,28,29,30]. *Y. lipolytica* cells exhibit more robust antioxidant mechanisms than other yeast species such as *Pichia pastoris* when exposed to OS induced by H_2_O_2_, Paraquat^®^, or heat [31]; in the same work, the authors observed that catalase was identified as the most active enzyme, followed by superoxide dismutase. In other studies, young *Y. lipolytica* cells exhibited diminished cell growth when exposed to pro-oxidant agents (H_2_O_2_, menadione, and juglone), whereas aged cells were more resistant to these treatments [32]. More recently, it was shown that aged *Y. lipolytica* cells exhibit up to a three times higher activity of catalase, superoxide dismutase, and glutathione peroxidase enzymes, when compared to young cells subjected to OS [33]. Despite these findings, the mechanisms regulating the antioxidant response in *Y. lipolytica* remain poorly understood, particularly in relation to OS resulting from environmental oxidizing agents or byproducts of its metabolism. In this study, we analyzed the expression patterns of genes encoding antioxidant enzymes in both young and aged *Y. lipolytica* cells exposed to OS conditions, to identify the gene(s) that play(s) a preponderant role in the antioxidant response and determine whether the antioxidant response-regulator mechanisms are modified in their different life stages.

## 2. Results

### 2.1. H_2_O_2_ Affects Y. lipolytica Cell Growth

**Figure 1:**
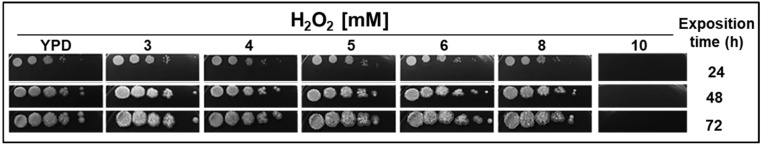
Effect of different H_2_O_2_ concentrations on *Yarrowia lipolytica* cell growth. Serial dilutions (1–1 × 10^−6^) of *Y. lipolytica* cells (P01a strain) growing in the middle of the logarithmic phase (OD = 1.0) were spotted in Yeast Peptone Dextrose (YPD) plates containing different H_2_O_2_ concentrations; then, plates were incubated at 28 °C and photographed every 24 h, at the indicated times.

Concentrations between 1 and 10 mM of H_2_O_2_ were tested, and the higher concentration was observed to affect the growth of *Yarrowia lipolytica* cells. Figure 1 shows the results obtained using different H_2_O_2_ concentrations [3–5 mM], which allowed cell duplication to continue (non-lethal dose). These could involve adaptation mechanisms that allow the survival of *Y. lipolytica* cells under oxidative conditions.

### 2.2. Y. lipolytica Cell Growth: Identification of Different Life Stages

**Figure 2:**
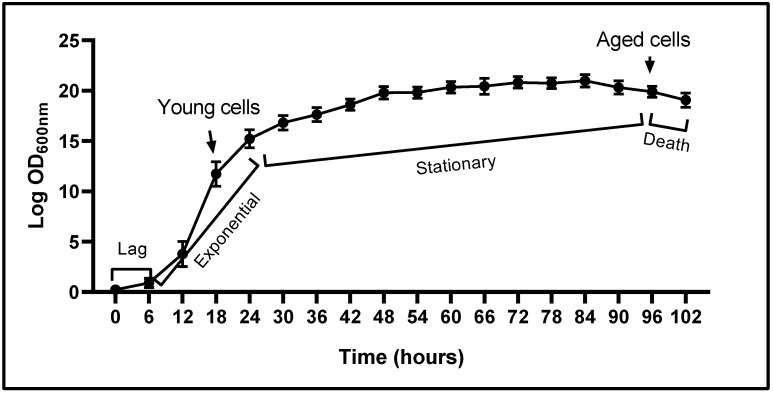
Growth curve of *Yarrowia lipolytica* P01a strain in YPD culture medium. *Y. lipolytica* cells were incubated at 28 °C and 200 rpm. OD_600_ values (average of three independent experiments) were determined at different times, as indicated (until 102 h). The figure shows the characteristic phases in cell growth pattern: phase lag, exponential growth, stationary phase, and the beginning of the death phase. Arrow indicated the culture times which cells (young and aged) were obtained.

The growth curve of *Y. lipolytica* cells, constructed with data corresponding to the OD_600_ values (Figure 2), shows a typical sigmoid pattern. There are four typical phases: lag, exponential (or logarithmic), stationary, and death. Our results showed that *Y. lipolytica* cells cultured in YPD medium were in the adaptive phase (phase lag) during the first 6 h of culture; between 6 and 24 h, they were in the exponential (log) phase (young cells, metabolically active); after 24 h, they began the stationary phase (yeast cell growth reaches a plateau); and at 96 h, the curve showed a light decay, which suggests the beginning of the death phase.

This growth pattern enabled us to identify the culture times corresponding to young (growing at the exponential phase, metabolically active, 12–24 h) and aged (growing at the end of the stationary phase, 96 h) *Y. lipolytica* cells.

### 2.3. Oxidative Conditions Modified the Gene Expression Profile

Changes in the expression profile of genes that code for the antioxidant enzyme in *Y. lipolytica* cells were analyzed using semi-quantitative RT-PCR, with specific primers designed for the nucleotide sequences corresponding to putative antioxidant genes obtained from databases (Table 1). The optimal conditions for each primer pair were determined experimentally (temperature gradient PCR), using genomic DNA as the template.

Cells were cultured until they reached the exponential phase; subsequently, the cells were submitted to the effect of H_2_O_2_ [5 mM] (oxidative conditions) or not (normal conditions), for different times (0–60 min). Then, the sample cells were processed, and gene expression analysis was performed. The *UBC6* (ubiquitin ligase) reference gene was used for value normalization in this process.

**Figure 3:**
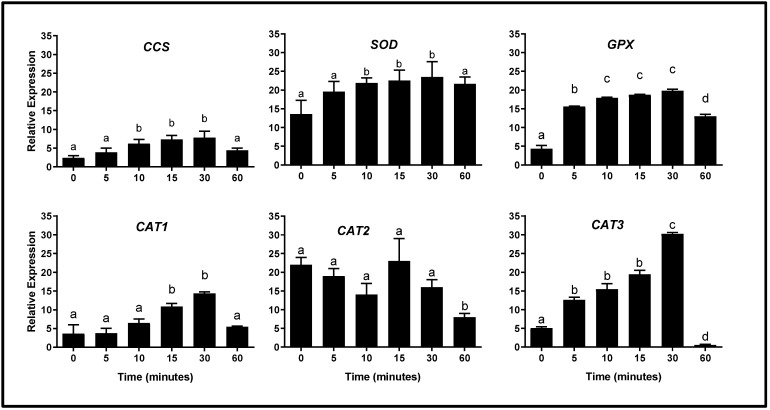
Expression profile of genes corresponding to the antioxidant system of *Y. lipolytica* cells subjected to oxidative conditions. *Y. lipolytica* cells growing in YPD medium (logarithmic phase) were exposed to hydrogen peroxide [5 mM] for different times, and gene expression analysis (by semi-qRT-PCR) was performed. Data are expressed as the mean ± standard deviation of three independent experiments performed in triplicate. As indicated in Section 4, for the gene expression analysis the PCRs were performed with 500 ng of cDNA and 22 PCR cycles; gene expression was normalized with respect to the constitutive *UBC6* gene. Different superscript letters (a, b, c, d) indicate statistically significant differences between time points (one-way ANOVA and Tukey’s post hoc test, *p* < 0.05). *CCS*: copper chaperone for Sod; *SOD*: superoxide dismutase gene; *GPX*: glutathione peroxidase gene; *CAT1*: catalase 1 gene; *CAT2*: catalase 2 gene; *CAT3*: catalase 3 gene.

Analyzing the genes that encoded enzymes corresponding to ADS of *Y. lipolytica* cells at different culture times in the presence of H_2_O_2_ [5 mM], this agent caused noticeable changes in their expression (Figure 3). The *CCS* gene (coding for Sod copper chaperone) showed a continuous increase (*p* < 0.05) in its expression, until it reached maximum values at 10–30 min after exposure to the oxidizing agent; however, at 60 min of exposure, the expression values returned to baseline levels. A similar pattern was also observed in the expression of the *SOD* gene. On the other hand, the *GPX* gene expression increased (*p* < 0.05) from 5 min to 30 min of exposure, which indicates a more accelerated response of this gene to the oxidizing agent; however, after 60 min of treatment, the expression of *GPX* decreased, but did not reach the baseline level of expression. It is possible that a longer incubation time is required, which could be related to a longer activity time of the enzyme encoded by this gene (the expression values are shown in Table 2).

For the genes that encoded for catalases, the *CAT1* gene expression values increased (*p* < 0.05) at 15–30 min of exposure to H_2_O_2_ while, at 60 min after exposure, the expression of this gene returned to the baseline values; however, it is interesting to note that the expression values were lower (considering the relative expression values observed in the other genes). The *CAT2* gene expression showed slight variations without presenting significant changes and, after 60 min of exposure, the values decreased below the baseline level.

On the other hand, this analysis showed that the transcription of the *CAT3* gene had a faster response to the oxidant environment. Its expression increased after 5 min of exposure to H_2_O_2_, maintaining this expression increase until 30 min of exposure (when the maximum expression peak of the *CAT3* gene was reached). At 60 min, the expression level decreased sharply to below the baseline value. The complete data corresponding to gene expression values in response to OS, are shown in Table S1 (Supplementary Materials).

### 2.4. The Gene Expression of the Antioxidant Response Is Higher in Young Y. lipolytica Cells

Analyzing the different genes of *Y. lipolytica* cells, the largest changes in gene expression were reached between 15 and 30 min after exposure to oxidative conditions. Considering these results, we analyzed the expression of genes that encode enzymes of the cellular antioxidant system, comparing the response of young cells (from logarithmic phase) and aged cells (stationary phase).

*Y. lipolytica* cells (P01a strain) were cultured in YPD media until the culture reached the logarithmic and stationary phases. Subsequently, H_2_O_2_ [5 mM] was added to each culture, the cells were cultured for 15 min and gene expression analysis was performed as described in Section 4.

The *CCS* and *SOD* genes showed no changes in their expression levels in young and aged cells. A similar result was observed with the *GPX* gene analysis (Table 2). These results suggest that both cell types have a similar transcriptional response to exposure to the oxidizing agent H_2_O_2_. With respect to the comparison of the expressions of genes encoding catalases (Table 2), both *CAT1* and *CAT2* genes have higher expression values in young cells than in the aged cells. On the other hand, the *CAT3* gene did not present significant expression changes in young and aged cells. Although there were no significant differences in the levels of expression of all the analyzed genes (except for the *CAT1* and *CAT2* genes), we can see that the expression levels were higher (except for *CAT3* gene) in the young *Y. lipolytica* cells (Table 2). Data corresponding to the comparisons of gene expression between young and older *Y. lipolytica* cells are shown as bar chart in Figure S1 (Supplementary Materials).

## 3. Discussion

Aerobic organisms are constantly exposed to reactive oxygen species (ROS), generated as byproducts of normal metabolism, especially through respiration. To modulate ROS concentrations and counteract their deleterious effects (are responsible to oxidative stress), there are several antioxidant systems with apparent functional redundancy, which can provide an evolutionary advantage.

To counteract OS effects, organisms have developed antioxidant defense mechanisms. Species such as *S. cerevisiae* possess multiple H_2_O_2_ detoxifying enzymes, such as catalases, superoxide dismutases, cytochrome C peroxidase, glutathione peroxidases, glutaredoxins, and peroxiredoxins, described as isoforms present in different cellular compartments.

The effects of several factors that increase ROS production and consequently induce OS in *Y. lipolytica* (pH, air pressure, temperature, chemical agents such as H_2_O_2_, menadione, and paraquat) have been well-described. The effects of these factors have an impact on the activity of the enzymes in the antioxidant defense system [31,32,33]. When *Y. lipolytica* cells in the exponential growth phase were exposed to H_2_O_2_ [50 mM], a lethal effect was observed, with a less than 1% survival rate of cells after treatment [34]. A similar phenomenon due to H_2_O_2_ was also observed in other yeast species; Kavitha and Chandra (2014) found that *Ashbya gossypii* cells submitted to the effect of H_2_O_2_ [10–25 mM] did not stop growing, but the growth rate decreased [35].

In previous studies by our research group, it was observed that H_2_O_2_ [5 mM] delayed *Y. lipolytica* cell growth without compromising its viability [36]. Studies with *S. cerevisiae* and *Candida oleophila* showed similar results [37,38,39].

The present findings suggest that *Y. lipolytica* cells, when subjected to H_2_O_2_ [5 mM], are able to develop defense mechanisms and adapt to OS conditions produced by this pro-oxidant compound. This adaptation is evidenced by changes in the expression levels of genes encoding enzymes involved in the cellular antioxidant response.

Also, our results show that exposure to oxidative conditions—namely, generated by H_2_O_2_ [5 mM]—leads to changes in the response of several antioxidant defense genes. A notable increase in the expression levels of the *CCS*, *SOD*, *GPX*, and *CAT3* genes was observed. Such results suggest that the antioxidant response in *Y. lipolytica* cells is regulated at the transcriptional level. It is interesting to note that the expression levels of the genes showed a trend toward recovery from homeostasis with respect to the exposure time (the expression levels decreased towards the end of H_2_O_2_ exposure); this observation can be explained due to the high adaptability to stress by yeasts. It has been mentioned that exposing an organism to mild stress to increase its tolerance to much stronger stress can induce cross protection against a variety of abiotic stresses in eukaryotic microorganisms [40,41].

Regarding the function of catalases (enzymes responsible for degrading H_2_O_2_ into H_2_O and O_2_), Gao et al. (2019) noted that induction of catalase activity is critical for cell protection against exogenous H_2_O_2_, to ensure cell survival [42]. Our results show that the expression of the *CAT1* and *CAT2* genes not increased as the time of exposure to H_2_O_2_ extended, consistent with previous findings, as cytosolic catalase (encoded by *CAT1* and *CAT2* genes) plays a primary role in cell survival under OS, while peroxisomal catalase (identified in *Y. lipolytica* as *CAT3*) is essential to adapt to this hostile environment, as described by Vázquez et al. [43].

In contrast, the expression of genes such as *SOD* increased within a few min of exposure to the oxidizing agent. It has been reported that the exposure of yeast cells to H_2_O_2_ [0.4 mM] for 20 min is sufficient to “awaken” a response in the ADS. Despite the fact that its main function is in the cytoplasm, the protein encoded by *SOD* performs a dual function as it has been reported that, when the level of H_2_O_2_ increases, Sod rapidly enters the nucleus and its activity is increased to avoid damage to DNA. Unlike the very short-lived superoxide free radical, H_2_O_2_ has a long half-life that allows it to diffuse into the nucleus and cause damage to genomic DNA; therefore, H_2_O_2_ is assumed to be the key ROS signaling that controls the nuclear translocation of Sod to prevent oxidative damage [44].

Changes in the expression levels of the *CCS* gene (which encodes a copper/zinc chaperone for Sod) were observed. Although the precise chemical mechanism is not yet certain, it is hypothesized that this chaperone is involved in mediating the post-translational regulation of Sod in response to increases in OS [45,46]. This could explain the prompt response in increasing the expression of the *CCS* gene in *Y. lipolytica* cells, although the expression levels for this gene were not the highest observed.

With respect to the *GPX* gene, the main function of glutathione peroxidase (encoded by the *GPX* gene) is to maintain the stability of mitochondrial membranes, protecting lipids from peroxidation and reducing the excessive production of ROS due to aging. In yeast, Gpx is localized in the mitochondria and reduces both inorganic and organic peroxides; it has been proposed that the absence of polyunsaturated fatty acids (PUFA) in yeast membranes may mask the antioxidant role of Gpx in mitochondria [47]. At the transcriptional level, we observed that *GPX* gene expression in *Y. lipolytica* cells reached the highest levels (with respect to the other analyzed genes). The increase in expression was immediate (5 min post treatment). These results suggest that under the oxidative conditions (due to the H_2_O_2_ [5 mM]) used in this work, the Gpx protein (encoded by *GPX* gene) is active, and its regulation occurs at the transcriptional level, contrary to the report by Shao et al. [22].

Vázquez et al. (2017) analyzed the effect of H_2_O_2_ according to cell growth phases. The authors described that *S. cerevisiae* cells growing in the exponential phase were more susceptible to stress than cells growing in the late stationary phase [48].

In young cells, the molecular response to ambient conditions is more robust [49]. In yeast cells, Zhang et al. (2023) described that Yap1, which is a key transcription factor responding to OS, was found to be temporally and highly activated in the early age and fast-dividing subgroup [27]. In this work, cells (young and aged) of *Y. lipolytica* were exposed to oxidative conditions and, when we analyzed and compared the expression pattern of the ADS genes, it was evident that the highest expression levels (for all the analyzed genes) were reached in the young cells.

In the comparison between the antioxidant response of young and aged *Y. lipolytica* cells, our results showed a higher expression of genes coding enzymes of the ADS in the young cells. Previous studies comparing the antioxidant response of young and aged *Y. lipolytica* cells have stated that in aged cells subjected to OS, the enzymatic activity of catalases, superoxide dismutases, and glutathione peroxidases increased up to two times [33]. In addition, young cells’ growth was diminished in the presence of pro-oxidant agents, with respect to that observed in aged cells [50].

It is necessary to mention that, although these works analyzed the cellular response to the presence of oxidizing agents (including H_2_O_2_), the analyzed parameters were enzymatic activity and cell growth. In addition, in the work of Arinbasarova et al. (2015), the activity of antioxidant enzymes was compared between cells (growing in exponential and stationary phases) without oxidative treatment [33]. On the other hand, in Biryukova’s work [50], *Y. lipolytica* cells were pretreated with a low concentration [0.5 mM] of H_2_O_2_, before being submitted to the effect of H_2_O_2_ [120 mM]. It is possible that, as has been reported, pretreatment with a low H_2_O_2_ concentration stimulated the response of the ADS; consequently, the exposed cells were more resistant and showed higher growth.

Further research efforts might aim to identify possible changes in the expression pattern of *Y. lipolytica* ADS genes under different oxidative conditions, for example, preconditioning cells to the effect of H_2_O_2_ or comparing the antioxidant response to different pro-oxidant agents.

## 4. Materials and Methods

### 4.1. Identification of Sequences That Encode the Genes of Interest

Gene sequences were obtained from the databases of the National Center for Information and Biotechnology (NCBI; https://www.ncbi.nlm.nih.gov/, accessed on 23 May 2025) and Universal Protein Resource (UniProt; http://www.uniprot.org/, accessed on 23 May 2025). Exons and introns were identified using the ORF-FINDER (https://www.ncbi.nlm.nih.gov/orffinder/, accessed on 23 May 2025) search tool to select the coding regions. After identifying the sequences, specific oligonucleotides were designed for the analysis of gene expression using reverse transcriptase–polymerase chain reaction (semiquantitative RT-PCR).

### 4.2. Microorganisms and Culture Conditions

The *Y. lipolytica* strain used in this work was P01a (MatA, Leu2-270, Ura3-302), provided by C. Gaillardin. This strain was cultured in solid or liquid YPD medium (1% yeast extract, 2% peptone, 2% glucose, and 2% agar when required). Cultures were kept overnight at 28 °C under shaking (200 rpm).

For cultures in different conditions, yeast cells growing in the exponential phase were inoculated (cell density OD_600_ = 0.2) in 250 mL Erlenmeyer flasks containing 50 mL of YPD liquid medium; cultures were incubated at 28 °C under shaking conditions (200 rpm).

### 4.3. Yarrowia lipolytica Growth Curve

The growth rate was determined by measuring the cell density OD_600_ (spectrophotometer Evolution 300, Thermo Scientific, Waltham, MA, USA) every 6 h, until 102 h. The results obtained were plotted to generate a growth curve that enabled us to determine the culture times corresponding to the different cell cycle stages of *Y. lipolytica*.

### 4.4. Effect of Different H_2_O_2_ Concentrations on Cell Growth

To determine the concentrations of H_2_O_2_ that inhibited the growth of *Y. lipolytica* cells, the serial dilution plate method [51,52,53] was used. Accordingly, tenfold serial dilutions (cell density OD_600_ = 1, until 1 × 10^−6^) of a suspension of *Y. lipolytica* cells growing in the logarithmic phase were prepared, and 3 μL of each dilution was spotted on YPD plates, added—or not—with different concentrations of H_2_O_2_ [1–10 mM]. Subsequently, the plates were covered with aluminum foil to avoid light exposure, incubated upside down at 28 °C, and photographed (Gel Doc-It Imaging System, UVP, Upland, CA, USA) every 24 h, for 3 days.

### 4.5. Oxidative Stress Induction and Nucleic Acid Extraction

To induce OS, *Y. lipolytica* cells growing in the logarithmic (young cells) or stationary (aged cells) phases were submitted to the effect of H_2_O_2_ [5 mM], during different times (0 to 60 min). A culture control was performed in YPD liquid medium without H_2_O_2_. At the corresponding times, cells were harvested, and the isolation of nucleic acids (DNA and RNA) was carried out following the glass bead lysis protocol described by Hoffman and Winston, which combines chemical and mechanical methods for cellular lysis [54]. The obtained nucleic acids were quantified by measuring their OD at 260 nm (Nanodrop 2000 UV–Visible, Thermo Scientific, Waltham, MA, USA). The sample integrity was determined via electrophoresis in 0.7% agarose gels.

### 4.6. mRNA Purification and cDNA Synthesis

DNA was removed from the nucleic acid samples by treatment with the enzyme DNase I PureLink (Invitrogen, Waltham, MA, USA), following the manufacturer’s instructions. Briefly, samples (1000–1500 ng) of nucleic acid were incubated with 1 μL (ca. 3 enzymatic units) of the enzyme for 1 h, at 37 °C; then, to inactivate the enzyme, 1 μL EDTA (25 mM) was added, and the mixture was incubated at 65 °C for 10 min. The concentration of RNA in the DNase-treated samples was adjusted to 100 ng/μL with DEPC (diethyl pyrocarbonate)–water. The final concentration of the RNA samples was determined using a NanoDrop ND-1000 spectrophotometer™ (Thermo Scientific, Waltham, MA, USA). For cDNA synthesis, the GoScript reverse transcription system (Promega, Madison, WI, USA) was used, following the manufacturer’s instructions. For each reverse transcription reaction, 400 ng of RNA and oligo dT (0.5 µg) were used.

### 4.7. Gene Expression Analysis

Transcriptional analysis was performed using semi quantitative RT-PCR. Polymerase chain reactions (PCR) were performed using conventional methodology, using the cDNA produced by reverse transcription, DNA polymerase (MyTaq, Bioline, London, UK), and specific primers (Table 1) corresponding to the genes that code for the antioxidant enzyme superoxide dismutase and Sod copper chaperone (YALI0E12133g and YALI0F30877g), catalases (YALI0E34265g, YALI0E34749g, and YALI0F30877g) and glutathione peroxidase (YALI0E02310g) of *Y. lipolytica*. When the semi-quantitative RT-PCR method is used for gene expression analysis, critical parameters are the use of a determined number of PCR cycles that enable noting the differences between the expression gene in the different probed samples, and the use of a constitutive gene used to the normalization of results in the different experiments. For the determination of this parameter, the amplification of 3 different constitutive genes (*ACT*, encoding actin; *UBC6*, encoding ubiquitin-conjugating enzyme; and *ALG9*, encoding a mannosyl-transferase) was performed using cDNA obtained as described in Section 4.6. Based in our results the *UBC6* gene (encoding ubiquitin-conjugating enzyme) was chosen as constitutive gen due that of all tested genes, this gene shown to be more stable in its expression values (*p* < 0.92) in all tested conditions (oxidative conditions). In addition, the *UBC6* gene was been proposed as constitutive gen by Teste et al. (2009) [55]. For the RT-PCR process standardization, different conditions were examined: different amounts of cDNA (50, 100, 200, 500, and 1000 ng) and different numbers of cycles of PCR (15, 20, 22, 25, and 32 cycles). Finally, considering the results obtained from these experimental approaches, the PCRs for the expression analysis were performed under the following conditions: denaturing, 95 °C, 30 s; annealing, 60 °C (as indicated in Table 1); extension, 60 s (enough for the amplification of the different PCR amplicons, whose size is shown in Table 1). The PCRs were performed in a Thermocycler (Sprint Thermal Cycler, Thermo Electron Corporation, Waltham, MA, USA), with 500 ng of cDNA and 22 PCR cycles for the expression analysis of each gene, using the constitutive *UBC6* gene to normalize the data obtained from each experiment. PCR products were resolved using electrophoresis in 2.5% agarose gels, stained with ethidium bromide, and imaged using the GelDoc-It Imaging System UVP analyzer (UVP; Upland, CA, USA). Densitometry was performed using Launch VisionWorks LS software, version 7.0.1 (UVP; Upland, CA, USA). The specific gene expression was normalized to the *UBC6* constitutive gene. During the process of gene expression analysis, a rigorous control of each step was done; this quality control in the different steps of the RT-PCR protocol can see in Figure S3 (Supplementary Materials). Changes in the expression levels of the different genes in cell types and conditions were calculated.

### 4.8. Statistical Analysis

All experiments were performed in triplicate, and the data are expressed as the mean ± standard deviation, unless otherwise indicated. Statistical analysis was performed using ANOVA one-way or Student *t* tests with the statistical package SPSS v21.0. The statistical significance was determined at *p* ≤ 0.005.

## 5. Conclusions

This study identified at least one gene encoding copper/zinc chaperone for Sod (*CCS* gene), one gene encoding superoxide dismutase (*SOD*), one gene encoding glutathione peroxidase (*GPX*), and three genes encoding catalases (*CAT1*, *CAT2*, and *CAT3*) in the genome of *Y. lipolytica*. The results suggest that the enzymes encoded by these genes are regulated at the transcriptional level and play pivotal roles in the organism’s antioxidant response. Also, at the transcriptional level, the antioxidant response of *Y. lipolytica* reached the maximal values at 10 to 15 min of oxidative treatment, which suggested a fast adaptive process to OS in this species. Notably, this response appears to be unaffected by cellular aging, although expression levels of these genes are lower in aged cells. To the best of our knowledge, this is the first study to demonstrate that the antioxidant response in *Y. lipolytica* is regulated at the transcriptional level, showing the expression pattern of each gene in response to the time of exposure to oxidative conditions.

## Figures and Tables

**Table 1:** Primer specifications and conditions used for semi-quantitative reverse transcriptase–polymerase chain reaction.

Gene	NCBI-RS	Primer Sequence (5′→3′)	Annealing Temperature (°C)	Amplicon Size (bp)
*CCS*	YALI0F30877g	F: CACTGGAACTTCTGCTCCGTCCC R: CTGGACGTCCTTTCGCTCCTCC	60	547
*SOD*	YALI0E12133g	F: CTTCGAGGAGATTCCAAGGTCTCC R: CTTGAGAGAGTCGGCATGGCC	60	390
*GPX*	YALI0E02310g	F: CCGCTTTCTACAACCTCGCTCC R: CGACGTTACCGTGCTTATCAACC	60	411
*CAT1*	YALI0E34265g	F: CCACCACCGTGCGATTTTCTACC R: CATGGTCTGAAGGGAAACGGTCC	60	539
*CAT2*	YALI0E34749g	F: CCATGCAAAGGGAGGAGGAGCC R: CCGTCCACGAGGGGTAATCCC	60	623
*CAT3*	YALI0F30987g	F: CAAGACCTTCACTCGATTCTCCACC R: CGTCATTGGTGAGGTTCTTGATGCC	60	425
*UBC6*	YALI0E30173g	F: CCGCGAAACCAGCAGGAACAATCTCC R: CCGAGGAATCTAGCTGCCACAATCC	60	598

NCBI-RS: Reference sequences from the National Center for Biotechnology Information of *Yarrowia lipolytica* genes: Sod copper chaperone (*CCS*), superoxide dismutase (*SOD*), glutathione peroxidase (*GPX*), catalase 1 (*CAT1*), catalase 2 (*CAT2*), catalase 3 (*CAT3*), and ubiquitin-conjugating enzyme (*UBC6*). F: forward primer, R: reverse primer, °C: degrees Celsius, bp: base pairs.

**Table 2:** Expression of antioxidants genes in young and aged cells of *Yarrowia lipolytica* subjected to H_2_O_2_ treatment.

Gene	Young Cells	Aged Cells	*p*-Value
*CCS*	7.66 ± 5.22	4.43 ± 1.13	0.2695
*SOD*	26.09 ± 2.76	16.97 ± 5.24	0.0800
*GPX*	19.35 ± 7.13	13.20 ± 6.10	0.1818
*CAT1*	13.81 ± 2.97 *	6.48 ± 3.06 *	0.005
*CAT2*	23.86 ± 2.37 **	11.30 ± 2.54 **	0.0001
*CAT3*	27.62 ± 4.92	27.77 ± 1.80	0.9595

Both young (logarithmic phase) and aged (stationary phase) *Y. lipolytica* cells were cultured in YPD medium containing H_2_O_2_ [5 mM] for 15 min. Data correspond to mean ± standard deviation of three independent experiments. For the gene expression analysis, the PCRs were performed with 500 ng of cDNA and 22 PCR cycles; gene expression was normalized with respect to the constitutive *UBC6* gene. * Indicates statistically significant differences between groups (*p* ≤ 0.005, Student’s *t*-test); ** (*p* ≤ 0.0001, Student’s *t*-test). *CCS*: encoding gene to copper chaperone for Sod gene; *SOD*: superoxide dismutase gene; *GPX*: glutathione peroxidase gene; *CAT1*: catalase 1 gene; *CAT2*: catalase 2 gene; *CAT3*: catalase 3 gene.

## Data Availability

The original contributions presented in this study are included in the article and Supplementary Materials. Further inquiries can be directed to the corresponding author.

## Supplementary Materials

The following supporting information can be downloaded at: https://www.mdpi.com/article/10.3390/ijms26199629/s1.
