# Supplementary material for: Antioxidant System Response of Yarrowia lipolytica Cells Under Oxidative Stress
Source: Int J Mol Sci. 2025 Oct 2;26(19):9629. doi: 10.3390/ijms26199629 (PMC12525467; doi:10.3390/ijms26199629)
Supplement: Supplementary file 1 [file ijms-26-09629-s001.zip › ijms-3856215-supplementary.pdf]

Supplementary material. (Manuscript ID: ijms-3856215).

Table S1. Expression profile of genes related to the antioxidant system in *Yarrowia lipolytica* cells exposed to oxidative stress induced by H_2O_2

Time (m)	*CCS*	*SOD*	*GPX*	*CAT1*	*CAT2*	*CAT3*
0	2.4±0.6^{a}	5.9±1.6^{a}	4.3±0.9^{a}	0.036±0.024^{a}	2.8±0.2^{a}	2.2±0.2^{a}
5	3.9±1.1^{a}	8.5±1.2^{a}	15.6±0.1^{b}	0.0371±0.013^{a}	7±0.4^{a}	8.6±0.1^{b}
10	6.2±1.1^{b}	9.5±0.6^{b}	17.9±0.2^{c}	0.0646±0.01^{a}	8.6±0.8^{a}	12±0.1^{b}
15	7.3±1.1^{b}	9.8±1.2^{b}	18.7±0.2^{c}	0.1085±0.008^{b}	10.8±0.6^{a}	12.9±0.2^{b}
30	7.8±1.7^{b}	10.2±1.8^{b}	19.8±0.4^{c}	0.1433±0.004^{b}	16.8±0.2^{a}	6.2±0.3^{c}
60	4.4±0.6^{a}	9.4±0.8^{a}	13±0.5^{d}	0.0548±0.001^{a}	0.3±0.1^{b}	5.4±0.7^{d}

Yarrowia lipolytica cells growing in the logarithmic phase were cultured in YPD medium containing H_2O_2 [5 mM] during different times (0-60 min). Data are expressed as mean ± standard deviation of three independent experiments performed in triplicate. Gene expression was normalized using the *UBC6* reference gene. Different superscript letters (a, b, c, d) indicate statistically significant differences between time points (one-way ANOVA and Tukey's post hoc test, $p < 0.05$). *CCS*: copper chaperone for Sod1 gene; *SOD1*: superoxide dismutase 1 gene; *GPX*: glutathione peroxidase gene; *CAT1*: catalase 1 gene; *CAT2*: catalase 2 gene; *CAT3*: catalase 3 gene.

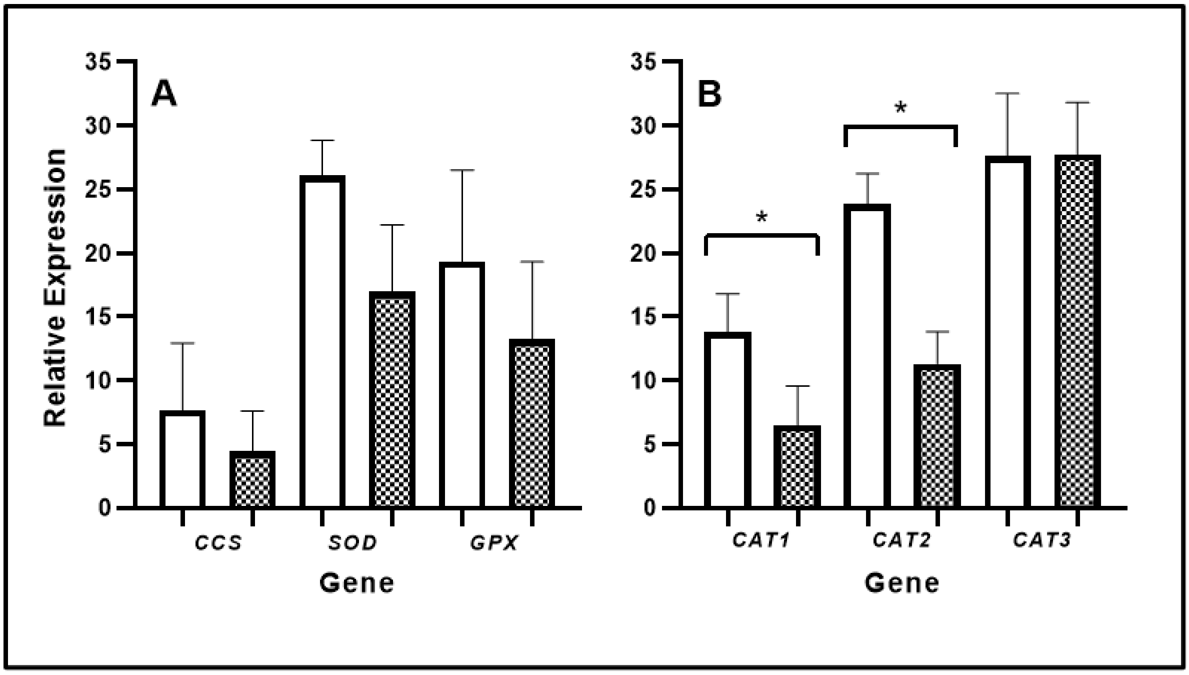

Figure S2. Expression levels of (A) *CCS, SOD,* and *GPX* genes, and (B) *CAT1, CAT2,* and *CAT3* genes, in young and aged *Y. lipolityca* cells subjected to H_2O_2 treatment. Both young (from logarithmic phase) and aged (stationary phase) cells were cultured in YPD medium containing H_2O_2 [5 mM] for 15 min. Data correspond to the mean ± standard deviation of three independent experiments (in triplicate; from each experiment, three samples were analyzed). Expression data were normalized with respect to the expression of the constitutive *UBC6* gene. White columns, young cells; dotted columns, aged cells. * indicates statistically significant differences between groups (p

< 0.05, Student's t-test). *CCS*: Copper chaperone for Sod 1 gene; *SOD1*: superoxide dismutase 1 gene; *GPX*: glutathione peroxidase gene; *CAT1*: catalase 1 gene; *CAT2*: catalase 2 gene; *CAT3*: catalase 3 gene.

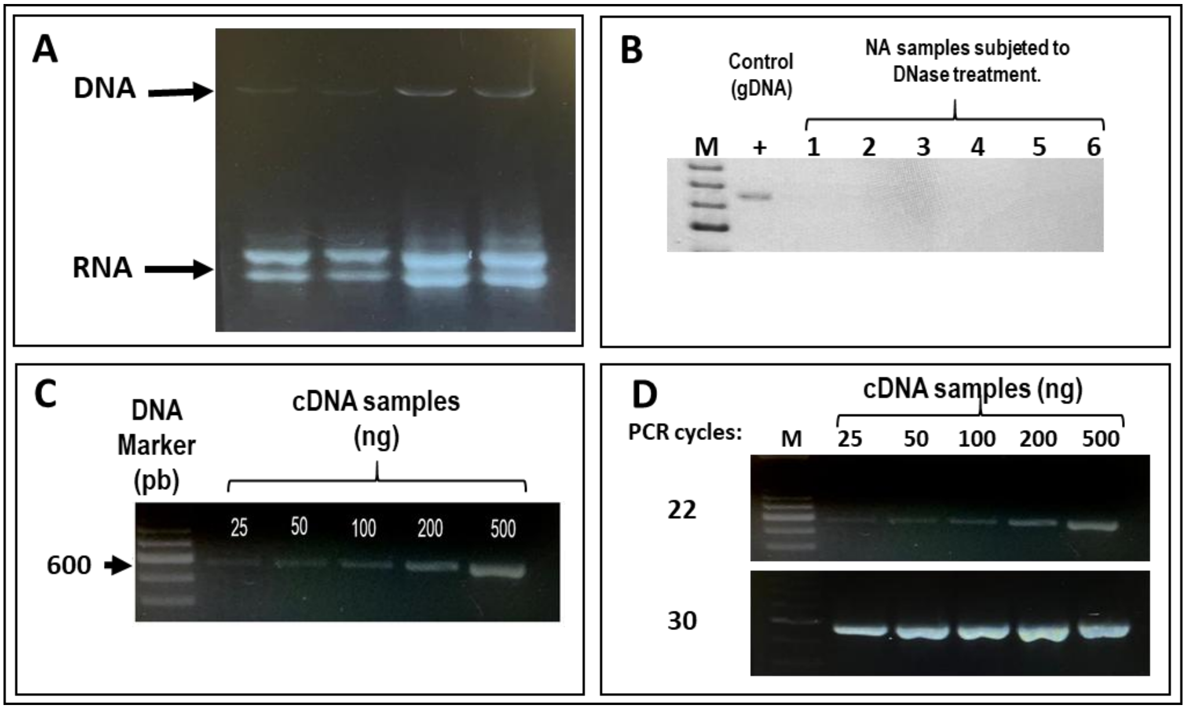

Figure S3. Conditions of the genetic material in different steps of the RT-PCR protocol. A): Bands corresponding to DNA and RNA are visible. Nucleic acids (NA) were extracted from *Y. lipolytica* cells using the Protocol described by Hoffman and Winston (1987). **B)** PCR products using samples of NA subjeted to different treatments (Tx) with DNase; a genomic DNA (gDNA) sample was included as positive control (+). Amplification reactions were performed with specific *CAT2* primers. All tested DNase Tx. were effectives for the gDNA elimination. **C)** Post RT reactions, several cDNA concentrations were tested in PCR reactions using specific *UBC6* primers. The quantity of PCR products increase when the cDNA quantity is higher. **D)** Different PCR cycles were tested, using different cDNA quantities. Images show only PCR results using 22 or 30 PCR cycles.
